# Treatment of Crohn’s disease-related high perianal fistulas combining the mucosa advancement flap with platelet-rich plasma: a pilot study

**DOI:** 10.1007/s10151-015-1311-8

**Published:** 2015-05-15

**Authors:** K. W. A. Göttgens, R. R. Smeets, L. P. S. Stassen, G. L. Beets, M. Pierik, S. O. Breukink

**Affiliations:** Department of Surgery and Colorectal Surgery, Maastricht University Medical Center, Post box 5800, 6202 AZ Maastricht, The Netherlands; Division of Gastroenterology-Hepatology, Maastricht University Medical Center, Maastricht, The Netherlands

**Keywords:** Mucosal advancement flap, Platelet-rich plasma, High perianal fistula, Recurrence, Crohn’s disease

## Abstract

**Background:**

Healing rates after surgical closure for high perianal fistula in patients with Crohn’s disease are even more disappointing than in patients with cryptoglandular fistulas. The objective was to improve healing rates by combining the well-known mucosal advancement flap with platelet-rich plasma.

**Methods:**

A prospective pilot study was conducted in one tertiary referral centre. Consecutive patients with primary or recurrent Crohn’s disease-related high perianal fistulas, defined as involving the middle and/or upper third parts of the anal sphincter complex, were included. A staged procedure was performed with non-cutting seton treatment for 3 months first, followed by a mucosal advancement flap with injection of platelet-rich plasma into the fistula tract.

**Results:**

Ten consecutive patients were operated on between 2009 and 2014. Half (50 %) of the patients had undergone previous fistula surgery. Mean follow-up was 23.3 months (SD 13.0). Healing of the fistula was 70 % (95 % confidence interval, 33–89 %) at 1 year. One (10 %) patient had a recurrence, and in two (20 %) patients, the fistula was persistent after treatment. An abscess occurred in one (10 %) patient. The median post-operative Vaizey score was 8.0 (range 0–21), indicating a moderate to severe continence impairment.

**Conclusions:**

The results of combining the mucosal advancement flap with platelet-rich plasma in patients with Crohn’s disease-related high perianal fistulas are moderate with a healing rate of 70 %. Further investigation is needed to determine the benefits and risks on continence status for this technique in this patient population.

## Introduction

High perianal fistulas (HPFs) are difficult to treat, and many techniques have been developed in recent years to reduce recurrence rates and to maintain optimal post-operative continence status [1–6]. Up to now, there is no consensus regarding the best technique for the treatment of this disease.

The high cryptoglandular perianal fistulas (HCPF) and the Crohn’s disease-related high perianal fistulas (CDRF) are the most common subtypes of HPF.

In a population-based cohort study, the cumulative frequency of perianal Crohn’s disease (CD) complications was 12 % at 1 year, 15 % at 5 years, 21 % at 10 years and 26 % at 20 years, and other population-based studies report incidence rates from 20 to 28 % [7–10]. According to the European Crohn and Colitis Organisation (ECCO) guidelines for complex perianal fistula, drainage of all abscesses, seton placement and dilatation of strictures are recommended first. Active luminal disease should be treated. Thiopurines in combination with antibiotics are the first medical choice [11]. Infliximab or adalimumab should be used as a second-line medical treatment [12–15]. Combining anti-tumour necrosis factor (TNF) treatment with ciprofloxacin may improve the outcome [16]. The recurrence rate of complex fistula after medical treatment is high, and therefore, combination with surgery is recommended. No surgery should be performed if active proctitis is still present. Similar surgical techniques are used for both HCPF and CDRF. However, healing rates are lower for CDRF. For example, the mucosal advancement flap (MAF), one of the most frequently used techniques, shows healing rates of about 60–80 % for HCPF compared to only 40–50 % for CDRF [17–20].

Other techniques for closure of CDRF show similar disappointing results, with long-term healings rates of around 55 % for fistula plugs [21], about 40 % for fibrin glue [22] and about 33 % after ligation of the intersphincteric fistula tract (LIFT) [23].

We have developed a technique in which the MAF is combined with the injection of platelet-rich plasma (PRP) into the fistula tract. PRP is hypothesized to improve wound healing and might improve fistula closure of HPF. Compared to fibrin glue, PRP in addition to clotting releases many growth factors, which are not present in fibrin glue. Long-term results using this technique for treatment of HCPF were previously published and show favourable results with healing of the fistula after 2 years of 83 % (95 % confidence interval (CI) 62–93 %) [3]. We hypothesized that the MAF in combination with PRP can also improve the outcome of complex CDRF. To the best of our knowledge, this treatment regimen has not been studied in CD patients before. We therefore performed an open-label prospective pilot study in primary and recurrent CDRF.

## Materials and methods

Between November 2009 and March 2014, 10 consecutive patients with primary or recurrent CDRF were included in this pilot feasibility study. HPFs were defined as fistula involving the middle and/or upper one-third of the anal sphincter complex. Recto-vaginal fistulas were excluded. Initial assessment of the fistula was done with clinical examination and magnetic resonance imaging (MRI). MRI was used to confirm a HPF and to classify the route of the fistula tract. Patients were only deemed fit for surgery if the luminal CD was in clinical and endoscopic (mucosal) remission after medical treatment according to ECCO guidelines.

The first part of the surgical procedure included non-cutting seton treatment for at least 3 months to reduce inflammation and drain sepsis, followed by a MAF with injection of PRP in the fistula tract. Patients on corticosteroids were first tapered off this medication. Patients with HPF of not due to CD were excluded, as well as patients with bleeding disorders, local or haematological malignancies and pregnant patients.

The primary outcomes of the study were healing and recurrence rates of the CDRF. The secondary outcome was continence status.

This study was conducted according to national medical ethical laws and guidelines, and written informed consent was obtained from all patients for the procedure and long-term follow-up in the outpatient clinic. The local medical ethics committee approved the study.

### Procedure and preparation of PRP

The surgical procedure and preparation of PRP were previously described for treatment of HCPF and were not changed for the treatment of CDRF in this study [3].

In short, patients were first treated with a non-cutting seton for drainage of the fistula tract and treated with a MAF combined with injection of PRP at least 3 months after placement of the seton. The PRP was made from 55 mL of the patients’ own blood, resulting in PRP with a 6–8 times higher concentration of platelets compared to baseline whole blood. A thrombin-coated syringe activated the PRP during injection into the fistula tract. The Gravitational Platelet Separation III (GPS-III) system instructions (Cell Factor Technologies, Biomet, Warsaw, IN, USA) were used for the preparation of the PRP.

### Follow-up

All patients were seen at the outpatient clinic for follow-up up to 1 year post-operatively. Follow-up visits were at 6 weeks, 3 months, 6 months and 1 year after surgery. If needed, patients were invited in between these follow-up visits. Fistula healing was defined as no more symptoms, a macroscopically closed external fistula opening and no drainage during manual compression. In case of doubt about closure, an MRI scan was performed to visualize a possible fistula tract. At the end of the study, patients who were not in clinical follow-up anymore were contacted by phone to check whether the fistula was closed. If this phone interview resulted in a suggestion of a recurrent fistula, the patient was invited to the outpatient clinic for physical examination. At the end of follow-up, the Vaizey score was used to evaluate continence status.

If the fistula was not closed 3 months after the operation, it was considered a persisting fistula or treatment failure. A new fistula occurring after a symptom-free period was defined as a recurrence.

## Results

Ten consecutive patients with CDRF were treated according to protocol and were followed up prospectively. There were three (30 %) males and seven (70 %) females. Median age was 47.5 years (range 30–67 years). Patient characteristics with previous treatments and study outcomes are shown in Tables 1 and 2. All patients were treated with a seton for at least 3 months first before the second operation was performed. All had a preoperative MRI scan. Five (50 %) patients had recurrent fistula.

**Table 1:** Patient characteristics and results

	Value
Male	3 (30 %)
Age	47.5 (30–67)
Smokers	4 (40 %)
BMI	25.7 (21.1–32.4)
Fistula location	Extrasphincteric: 1 (10 %) Intersphincteric: 2 (20 %) Transsphincteric: 7 (70 %)
Previous operations	None: 5 (50 %) One operation: 0 (0 %) Two operations: 1 (10 %) >Two operations: 3 (30 %) Unclear: 1 (10 %)
Recurrences	1 (10 %)
Persisting fistulas	2 (20 %)
Primary healing rate	7 (70 %)
Secondary healing rate	7 (70 %)

Values given as *n* (%) or as median (range)

**Table 2:** Patient history and outcome

Patient	Current treatment for Crohn’s disease	Fistula type	Previous fistula treatment	Stoma	Persisting fistula	Recurrence
1	Mesalazine; azathioprine	Primary	–	No	No	No
2	Adalimumab	Primary	–	No	No	Yes
3	Azathioprine; infliximab	Recurrent	Fistulotomy; seton treatment	No	No	No
4	Azathioprine; infliximab	Recurrent	Seton treatment (4×)	No	No	No
5	None	Recurrent	MAF, other treatments unknown	No	Yes	No
6	Infliximab	Recurrent	Seton treatment (3×), MAF + stem cells	No	No	No
7	Infliximab; 6-mercaptopurine	Primary	–	No	No	No
8	Adalimumab	Primary	–	No	No	No
9	Mesalazine; infliximab	Primary	–	No	Yes	No
10	6-Mercaptopurine	Recurrent	Seton treatment (3×), deviating colostomy	Yes	No	No

*MAF* mucosal advancement flap

Eight (80 %) patients’ CDRF healed, with a median healing time of 52.5 days (range 12–114 days), although one (10 %) patient showed delayed healing with a time to healing of 114 days (without any additional intervention). One (10 %) of these healed patients developed a recurrence 44 days after complete closure of the fistula. The other two (20 %) patients’ fistulas did not heal after the operation.

**Fig. 1:**
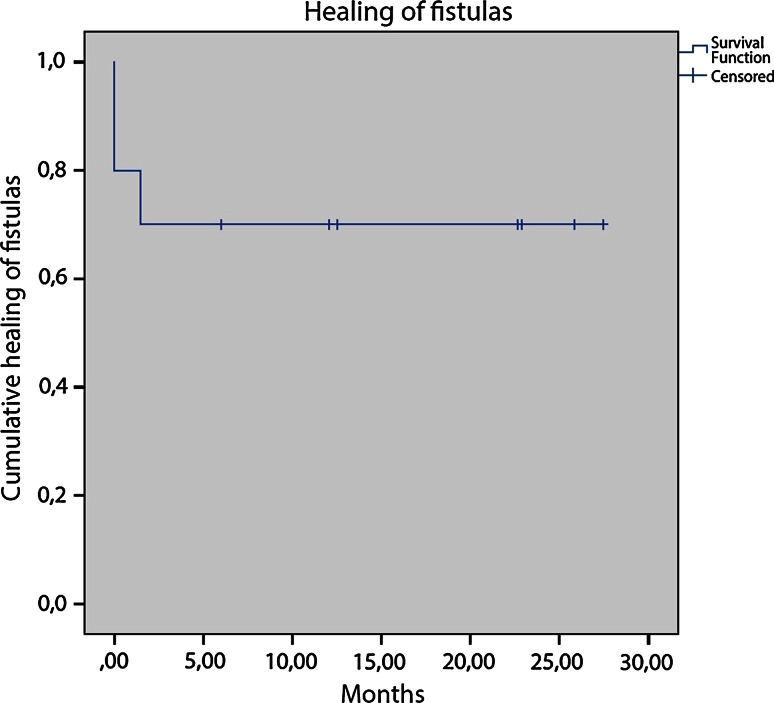
Healing of the fistulas. Patients at risk: 0 months: 8; 5 months: 7; 10 months: 5; 15 months: 3; 20 months: 2; 25 months: 2

A Kaplan–Meier curve was created to show healing of the fistulas (Fig. 1). Healing at 1 year was 70 % (95 % CI 33–89 %). Mean follow-up was 23.3 months (SD 13.0).

The patient with the recurrence was treated with a MAF + PRP again after another 3 months of seton treatment and developed another recurrence. One of the patients with a persisting fistula chose to have a colostomy and did not want other treatment for the CDRF. This fistula closed several months after colostomy placement. The other patient with a persisting fistula was treated with a MAF + PRP after another 3 months of seton treatment. This fistula is still not closed to date.

An abscess occurred in one (10 %) patient post-operatively. This was the patient with a persisting fistula, who later received a colostomy. No other complications occurred.

Seven (70 %) patients completed the Vaizey score questionnaire 6 months post-operatively. Two of these seven patients had a recurrence, and the others were all healed. The median Vaizey score was 8 (range 0–21). No preoperative data on continence status were available.

## Discussion

We report data of the first study combining the MAF with injection of PRP in the fistula tract for high CDRF. The healing rate was moderate with a healing of the fistulas at 1 year of 70 % (95 % CI 33–89 %). The median Vaizey score of 8.0 indicates a fairly severe impairment of continence status.

There were some limitations to this study. It was a small single-centre prospective pilot study to evaluate the effectiveness of adding PRP to the MAF in this patient group. Besides, data on preoperative continence status were not available, making it difficult to determine influence of the surgical procedure on continence status. Patients in follow-up for more than 1 year were evaluated using telephone interviews, which might have resulted in some bias in results.

As previously explained [3], our surgical procedure is based on the MAF, which is a well-known and much performed operation for HPF. The rationale behind using the MAF as the basis of our technique was to avoid long learning curves for surgeons and to make results reproducible. The results of our technique, as published previously, were promising for HCPF with healing at 2 years of 83 % (95 % CI 62–93 %) [3]. The results for the treatment of CDRF are less favourable than hoped, although our healing rates seem higher compared to the MAF alone [19, 20].

The reason for these less favourable results, compared to the treatment of HCPF, is not clear. However, it is known that the vascular endothelial growth factor (VEGF) response is defective [24], and platelet-derived growth factor (PDGF) might be responsible for maintenance of damaged vasculature in patients with CD [25]. Both growth factors have, respectively, a role in angiogenesis, and protein and collagen synthesis and are released when using PRP as described by van der Hagen et al. [26], thus improving wound healing. This, however, might not be true for patients with CD. Furthermore, platelets in patients with inflammatory bowel disease show higher levels of some interleukin receptors [27], which might change the effects of PRP. The function of PRP, concerning wound healing, in patients with CDRF might therefore be different. Unfortunately, no studies on the use of PRP in patients with CD are available.

Regarding continence impairment, it is difficult to draw conclusions. Our previous study in patients with HCPF did not show much impairment of continence status. This study in patients with CDRF resulted in a higher median Vaizey score of 8.0, which would be classified as major incontinence according to Dubsky et al. [28]. It is, however, shown that the prevalence of faecal incontinence in patients with CD is high, between 25 and 74 %, even without anal fistula surgery [29]. This would make it even more important to clarify the influence of our surgical procedure on, the perhaps already impaired, continence status of patients with CDRF.

Previous surgery, and specifically previous MAF, might also have had a significant influence on continence status. We use curettage for the fistula tract before performing the MAF, and other surgeons use only mild abrasive de-epithelialization or even resect and core-out the tract. Since all the previously treated patients were referred from others centres, we are unsure what the influence of the prior surgery was on their continence status.

## Conclusions

The healing rate of CDRF treated using our technique is 70 % and favourable compared to the 40–50 % for the MAF reported in other studies. Further investigation, preferably as a randomized study, into the usefulness of combining the MAF with PRP in patients with CDRF is needed to see whether healing rates can actually be improved, and especially to show the influence on continence status post-operatively in a patient population with an already high risk of faecal incontinence.
